# Inferior Frontal Gyrus-Based Resting-State Functional Connectivity and Medium Dispositional Use of Reappraisal Strategy

**DOI:** 10.3389/fnins.2021.681859

**Published:** 2021-06-17

**Authors:** Wenjuan Li, Ke Xie, Ronald K. Ngetich, Junjun Zhang, Zhenlan Jin, Ling Li

**Affiliations:** MOE Key Laboratory for Neuroinformation, High-Field Magnetic Resonance Brain Imaging Key Laboratory of Sichuan Province, Center for Psychiatry and Psychology, School of Life Sciences and Technology, University of Electronic Science and Technology of China, Chengdu, China

**Keywords:** emotion regulation, inferior frontal gyrus, prediction, resting-state functional connectivity, medium reappraisal

## Abstract

The previous neuroimaging functional connectivity analyses have indicated that the association between the inferior frontal gyrus (IFG) and other brain regions results in better emotion regulation in reappraisal tasks. However, no study has explored the relationship between IFG-based resting-state functional connectivity (rsFC) and the dispositional use of reappraisal strategy. Therefore, the present study examined the potential associations between rsFC patterns of both left and right IFG and dispositional reappraisal use. One hundred healthy participants completed the Emotion Regulation Questionnaire (ERQ) and underwent a resting-state functional magnetic resonance imaging (fMRI) acquisition. An approach of the seed-based rsFC analysis was recruited to estimate the functional connectivity maps of bilateral IFG with other brain regions, and the reappraisal scores from the ERQ were then correlated with the functional maps. Our findings showed that IFG-based rsFC was positively correlated with dispositional reappraisal only in the range of 4 to 5.5 points [medium reappraisal group (MRG)]. Specifically, medium dispositional reappraisal was positively correlated with rsFC between left/right IFG and bilateral temporal gyrus. Besides, medium dispositional reappraisal was positively correlated with rsFC between left IFG and bilateral superior parietal lobe (SPL), middle cingulate cortex (MCC), and right insula, as well as between right IFG and dorsomedial prefrontal cortex (DMPFC) and anterior cingulate cortex (ACC). In conclusion, these results indicate that bilateral IFG plays an important role in the medium use of the reappraisal strategy.

## Introduction

Effective emotion regulation is necessary for our daily social life. Essentially, various strategies can be employed to achieve successful emotion regulation, e.g., distraction, cognitive reappraisal, and expressive suppression (Webb et al., 2012; Morawetz et al., 2017b). Among these strategies, reappraisal, which entails the changing of the emotional value of stimuli that evokes emotions (Kanske et al., 2011; Webb et al., 2012), is the most frequently applied and studied strategy of emotion regulation (Wager et al., 2008; Kalisch, 2009; Ochsner et al., 2012; Buhle et al., 2014). Moreover, Gross and John (2003) developed a self-report Emotion Regulation Questionnaire (ERQ) to measure the dispositional use of two strategies, reappraisal (center on reinterpretation) and suppression. Assessment of this kind of personality habitude can reflect the utilization frequency of strategy, which may finally implicate the individual differences in abilities of emotion regulation. Also, more frequent use of reappraisal strategy has been demonstrated to be associated with better regulation of emotions, social interactions, and mental and physical health (Gross and John, 2003; Meyer et al., 2012; Hu et al., 2014; Picó-Pérez et al., 2018; Zaehringer et al., 2020).

The neural underpinnings related to reappraisal strategy have usually been evaluated by measurement of functional activation during experimental reappraisal tasks with functional magnetic resonance imaging (fMRI). The previous meta-analytic studies have shown that reappraisal recruits a widespread network that includes dorsomedial prefrontal cortex (DMPFC), dorsolateral prefrontal cortex (DLPFC)/superior frontal gyrus (SFG), ventrolateral prefrontal cortex (VLPFC)/inferior frontal gyrus (IFG), parietal lobes, temporal gyrus, and cingulate cortex (Phillips et al., 2008; Buhle et al., 2014; Kohn et al., 2014; Morawetz et al., 2017b). Importantly, the IFG/VLPFC is well known as a critical region for processes of selection and inhibition (Schulz et al., 2009; Kohn et al., 2014; Morawetz et al., 2017b), language (Ochsner et al., 2012; Messina et al., 2015), and social cognition (Kohn et al., 2014; Hartwigsen et al., 2019) in emotion regulation. In particular, the IFG has been observed with increased activation when multiple appropriate reinterpretations emerge and a choice must be made to achieve goal-directed behavior, as well as when required to inhibit goal-inappropriate reinterpretations (Morawetz et al., 2016; Braunstein et al., 2017).

Moreover, methods of IFG-based (with IFG serving as seed regions) functional connectivity have also been used to explore the neural correlations of reappraisal strategy in experimental settings. Morawetz et al. (2017a) defined left IFG as a seed region and examined effective connectivity between the seed region and the remaining brain regions, with reappraisal success scores as covariate. They have observed positive effective coupling between the left IFG and DLPFC, DMPFC, right middle temporal gyrus (MTG), and superior temporal gyrus (STG) during downregulation of emotion. Furthermore, in another study, Morawetz et al. (2016) found that the inhibitory effect on connectivity from IFG to DLPFC could facilitate successful reappraisal, deducing that the IFG may choose one from the many feasible goal-relevant reinterpretations actively maintained in the working memory (associated with DLPFC’s increased activity), and suppress the DLPFC as soon as the selection process is finished. On the other hand, Wager et al. (2008) found that the right IFG could effectively predict reappraisal success with some cortical and subcortical regions as mediators, such as DMPFC, SFG, inferior temporal gyrus (ITG), and subgenual anterior cingulate cortex (ACC).

Recent evidence has shown that the formation of intrinsic resting-state functional architecture is influenced by repeated task-based co-activation within a network (Mackey et al., 2013; Guerra-Carrillo et al., 2014; Uchida et al., 2014), suggesting a close correspondence between task-specific brain activation and intrinsic brain connectivity, which is reflected by resting-state functional connectivity (rsFC). In essence, Smith et al. (2009) compared task-based activation networks derived from a large database of functional imaging studies with the covarying networks from 36 subjects’ resting fMRI data, and found that these task-related networks closely matched the networks when at resting state. Intriguingly, another study using a sample of 4- to 18-year-old healthy participants found that task-related functional connectivity could even predict rsFC of up to 2 years after the initial experimental task (Gabard-Durnam et al., 2016). Thus, it seems possible that recurring activation caused by a specific task may share an association with resting-state connectivity pattern, and this may also apply to the emotion regulation domain with IFG-based functional connectivity during the reappraisal task. Besides, one study demonstrated that activation of IFG in reappraisal task is positively correlated with the frequency of dispositional reappraisal in daily life (Grecucci et al., 2013). Consequently, it can be assumed that the reappraisal task and dispositional reappraisal may share a similar IFG-based functional connectivity pattern. However, since no study has examined the association between dispositional use of reappraisal strategy and rsFC with IFG seed regions, it is uncertain whether IFG-based rsFC could also facilitate habitual reappraisal, thus resulting in better emotion regulation, although previous evidence indicates that both left and right IFG show associations with DMPFC and temporal gyrus during the performance of a reappraisal task. Nevertheless, it remains unknown whether the functional connectivity pattern of the left IFG seed concerning habitual reappraisal is the same as that of the right IFG seed.

Moreover, neural efficiency supposes that more adept individuals optimally use the functional connectivity to undertake minute neural processing and, hence, display diminished neural activity alongside the performance facilitation (Neubauer and Fink, 2009; Di Domenico et al., 2015; Curtin et al., 2019). It is anticipated that the higher the scores of dispositional reappraisal, the more frequent the use of reappraisal, and the better the ability of emotion regulation. Presumably, the neural efficiency may also be suitable for dispositional reappraisal, a daily used specific strategy of emotion regulation, with a changeable connectivity pattern along with a variation of reappraisal scores. However, there is no evidence supporting how the individual difference in frequency of reappraisal use may affect functional connectivity during the resting state. Considering the aforementioned close association between rsFC and task-related functional connectivity, we asked another question: Could it be possible that the IFG-based rsFC pattern vary with the level of frequency of dispositional reappraisal?

Therefore, in an endeavor to answer the questions raised, the present study applied seed-based rsFC analysis and prediction analysis with an aim of (1) examining whether IFG-based rsFC is related to individual dispositional use of reappraisal; (2) investigating whether habitual reappraisal related rsFC pattern of the left IFG is the same as that of the right IFG; (3) exploring whether IFG-based rsFC is specific to the frequency level of the use of dispositional reappraisal. Based on the limited evidence mentioned above, we hypothesized that dispositional use of reappraisal would be positively correlated with IFG-based rsFC and that the rsFC pattern of the left IFG and that of the right IFG would be very similar, with both showing associations with DMPFC and temporal gyrus. Moreover, according to the theory of neural efficiency (Neubauer and Fink, 2009; Di Domenico et al., 2015; Curtin et al., 2019), the individual differences in dispositional reappraisal may be associated with the difference in the IFG-based rsFC pattern, with a higher level of dispositional reappraisal corresponding to a less IFG-based rsFC.

## Materials and Methods

### Participants

One hundred and seven (*N* = 107) healthy, right-handed adults (59 females, 17 to 26 years old, mean age 21.36 ± 2.052 years) participated in the experiment after giving their written informed consent. All participants reported no history of mental disorders, head injury, or cardiovascular diseases. The study protocol was approved by the Local Committee for the Protection of Human Subjects of the University of Electronic Science and Technology of China and was conducted according to the declaration of Helsinki.

### Behavioral Assessment

All participants completed the ERQ (Gross and John, 2003) before scanning. The ERQ consisted of two subscales, cognitive reappraisal and expressive suppression. Ten items, with six for reappraisal and four for suppression, are included in the scale, whose choices ranged from strongly disagree (1) to strongly agree (7). In the current study, Cronbach’s α coefficient of reappraisal subscale was 0.828 and that of suppression subscale was 0.601. According to the scale used, four is the median score. In our interpretation, the individuals who scored less than four points do not frequently use either the reappraisal or suppression strategy. On the other hand, a score of more than four implies a frequent use of the aforementioned strategies. In our study, a very small number of participants (*n* = 7) recorded less than four points on the reappraisal subscale. Therefore, we found it appropriate to examine the intrinsic neural mechanisms of reappraisal strategy only among the individuals who frequently apply this strategy in their daily life. Hence, we excluded the data of *n* = 7 participants with low frequent use of reappraisal strategy (<4 points). Subsequently, we categorized the remaining *n* = 100 participants into two groups, according to their reappraisal scores. Those who had a score of between 4 (median) and 5.5 were classified as moderate users of reappraisal strategy and thus put into the medium reappraisal group (MRG). Similarly, those with a score of between 5.5 and 7 were considered as high-frequency reappraisal strategy users and, therefore, categorized into high reappraisal group (HRG). Ultimately, all the remaining participants (*n* = 100) were assigned into either MRG (*n* = 80) or HRG (*n* = 20). In MRG, neither scores of reappraisal and suppression nor age showed significant gender differences (all *p* > 0.08). This was the same case with HRG (all *p* > 0.4) (Table 1). On the other hand, the reappraisal score was higher than the suppression score in both groups [MRG: *t*_(79)_ = 11.989, *p* < 0.001; HRG: *t*_(19)_ = 11.995, *p* < 0.001]. Finally, to compare MRG with HRG, a subsample of 20 participants from MRG (named as sMRG) were selected, with similar age, gender, and suppression scores as HRG (all *p* > 0.4) (Table 2).

**TABLE 1:** Demographic characteristics and behavioral assessment.

	MRG				HRG			
	*n*	Age	Reappraisal	Suppression	*n*	Age	Reappraisal	Suppression
		M (SD)	M (SD)	M (SD)		M (SD)	M (SD)	M (SD)
Female	44	21.20 (2.11)	4.93 (0.35)	3.40 (0.91)	12	21.25 (1.82)	6.04 (0.44)	3.52 (0.89)
Male	36	21.58 (2.09)	4.92 (0.33)	3.76 (0.94)	8	21.25 (2.05)	6.21 (0.54)	3.50 (1.16)
*p*		0.425	0.891	0.088		1.000	0.460	0.964

*MRG, medium reappraisal group; HRG, high reappraisal group.*

**TABLE 2:** Comparison of behavioral assessment between HRG and sMRG.

	Gender	Age	Reappraisal	Suppression
	Female/Male	M (SD)	M (SD)	M (SD)
HRG	12/8	21.25 (1.86)	6.11 (0.48)	3.51 (0.98)
sMRG	12/8	21.70 (1.95)	4.90 (0.29)	3.43 (0.85)
*p*	1.000	0.460	<0.001	0.764

*HRG, high reappraisal group; sMRG, subsample of medium reappraisal group.*

### Image Acquisition and Data Analysis

A 3.0-T GE Sigma scanner was used to collect resting-state fMRI images with a gradient echo planar imaging (EPI) sequence (TR, 2,000 ms; TE, 30 ms; FA, 90°; FOV, 240 mm × 240 mm; matrix size, 64 × 64; voxel size, 3.75 mm × 3.75 mm × 3 mm; slices, 43). The T1-weighted structural image was acquired with a high-resolution T1-weighted scan (TR, 5.96 ms; TE, 1.96 ms; FA, 9°; FOV, 256 mm × 256 mm; matrix size, 256 × 256; voxel size, 1 mm × 1 mm × 1 mm; number of slices, 176). Participants were instructed to rest with their eyes closed but not to fall asleep during the scan.

Resting-state fMRI data analysis was conducted using the data processing assistant for resting-state fMRI toolbox (DPARSF^1^) and statistical parametric mapping software (SPM12^2^). To keep magnetic field stabilization, the first five EPI volumes of the fMRI images were removed. Preprocessing consisted of the following steps: Slice timing correction, 3D motion correction, nuisance covariates regression (Friston-24 motion parameters; white matter, cerebrospinal fluid, and global signals), spatial normalization to the Montreal Neurological Institute (MNI) template and resampling to 3 mm × 3 mm × 3 mm, removing the linear trends, temporal band-pass filtering (0.01–0.1 Hz), and spatial smoothing with a Gaussian kernel of full-width half-maximum 6 mm. The head motion exclusion was applied with translation not exceeding 3 mm and rotation not exceeding 3°, and mean FD_Jenkinson not exceeding 0.2 (Power et al., 2012; van Dijk et al., 2012). According to this threshold, no participant was excluded.

#### Seed Definition

**FIGURE 1:**
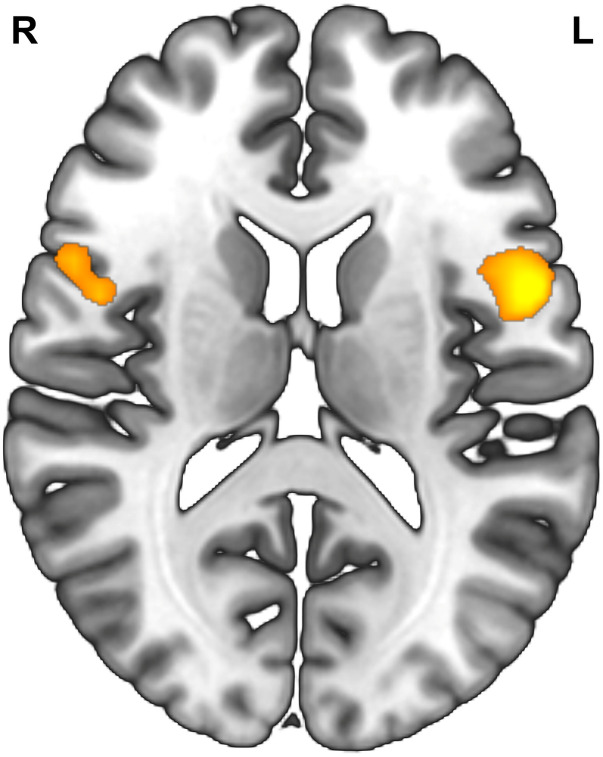
Result of voxel-wise functional connectivity. Brain regions whose degree centrality correlated with reappraisal scores. Statistical threshold of *P*_*uncorrected*_ < 0.005 was used for cluster correcting.

Degree centrality refers to the number of brain connections from a voxel to others across the whole brain (Zuo et al., 2012; Yan et al., 2017; Lv et al., 2018). The measure of degree centrality has been widely performed to examine node characteristics of intrinsic connectivity networks, especially for the identification of functional hubs in functional connectivity analysis (Zuo et al., 2012). In the present study, a voxel-wise functional connectivity analysis was performed using DPARSFA, and the examination of degree centrality was recruited to identify functional hubs that were related to individual reappraisal scores. The correlation threshold was set at *r* > 0.25 for the degree centrality calculation. Then, the resulting degree centrality was used to conduct a multiple regression analysis, with gender, age and suppression scores controlled as covariates of noninterest and reappraisal scores as a predictor of interest. For the exploratory purpose, we lowered the statistical threshold to *P*_*uncorrected*_ < 0.005 with a cluster size > 50 voxels. At this threshold, only two clusters emerged, the left and right IFG, whose degree centrality showed a positive correlation with reappraisal scores (Table 3 and Figure 1). Therefore, based on the results of the above voxel-wise functional connectivity analysis, left IFG (−48 9 6) and right IFG (54 12 18) were defined as seed regions for further seed-based rsFC analysis. The two seed regions were separately built as a 6-mm radius sphere centered around the peak activation using the Marsbar toolbox^3^.

**TABLE 3:** Results of voxel-wise functional connectivity.

Region	H	K	T	MNI coordinates		
				*x*	*y*	*z*
Inferior frontal gyrus	L	106	4.33	−54	6	15
			3.85	−45	9	18
			3.27	−51	12	3
Inferior frontal gyrus	R	69	3.85	45	12	9
			3.46	48	3	15
			3.42	54	12	15

*H, hemisphere; L, left; R, right; K, cluster size in number of activated voxels; T, T value; MNI, the Montreal Neurological Institute coordinates. Statistical threshold of *P*_*uncorrected*_ < 0.005 was used for cluster correcting.*

#### Seed-Based Voxel-Wise rsFC Analysis

After seeds extraction, seed-based voxel-wise rsFC analyses were performed to explore brain regions connected with left/right IFG, and the connectivity correlating with individual reappraisal scores. Firstly, time series of all voxels located within these two seed regions were abstracted and averaged, respectively. Secondly, a Pearson correlation was conducted between each seed region’s time series and those of all other brain voxels of each participant. Thereafter, the resulting correlation coefficients were transformed into Fisher’s *z* scores, representing the rsFC for each connection of each participant. Subsequently, multiple regression models were performed with reappraisal scores as a predictor of interest, and the effect of gender, age, and suppression scores simultaneously eliminated. All activations were applied at the whole-brain level with a statistical significance of false discovery rate *P*_*FDR*_ < 0.05 and a cluster extent > 50 voxels. Besides, multiple regression models were also performed with suppression scores as a predictor of interest, suggested by Picó-Pérez et al. (2018) in their similar study on the association between dispositional use of emotional regulation strategies and rsFC, but with the amygdala as seed regions. However, we did not observe any significant activation when we used the suppression scores as a predictor of interest at the same threshold of *P*_*FDR*_ < 0.05. Consequently, the suppression strategy was not included in the result.

In addition, we further extracted the rsFC strength value of each region of interest (ROI), which amounted to a sphere of 6-mm radius centered around the peak of activation using Marsbar toolbox (see text footnote 3). Then, the partial correlation analyses were performed between connectivity strength and reappraisal scores after controlling for gender, age, and suppression scores.

Initially, we tried to investigate the potential association between IFG-based rsFC and reappraisal scores across the entire range observed in the sample of *n* = 100. Unfortunately, we did not find any significant activation at the threshold of *P*_*FDR*_ < 0.05. The previous literature, especially the theory of neural efficiency, may provide a reasonable conjecture that individuals with a higher level of frequency of dispositional reappraisal use may show less connectivity between IFG and the remaining regions. Hence, we considered that, probably, the high-frequency level of dispositional use of reappraisal affects the association between IFG-based rsFC and reappraisal scores. Therefore, as reported before, the 100 participants were allocated into two groups (MRG and HRG) according to the range of reappraisal scores.

#### Prediction Analysis Using Cross-Validation

To test whether the observed functional brain features in MRG could reliably predict reappraisal scores of new individuals, internal cross-validation analyses were performed using the Pattern Recognition for Neuroimaging Toolbox (PRoNTo v2.1^4^). The input vectors were mean-centered using the training data (Xie et al., 2020), while the effect of covariates of noninterest (gender, age, and suppression scores) was regressed out. The predictive power was assessed by calculating Pearson’s correlation coefficients between the predicted and actual reappraisal scores. Additionally, the statistical significance of the correlation was determined by 5,000 times of permutation testing without replacement.

In order to examine whether the reappraisal related IFG-based rsFC pattern in MRG is the same as that in HRG, internal cross-validation analyses were conducted using the reappraisal related rsFC of sMRG to predict the reappraisal scores of HRG.

## Results

### Functional Connectivity Analysis

**FIGURE 2:**
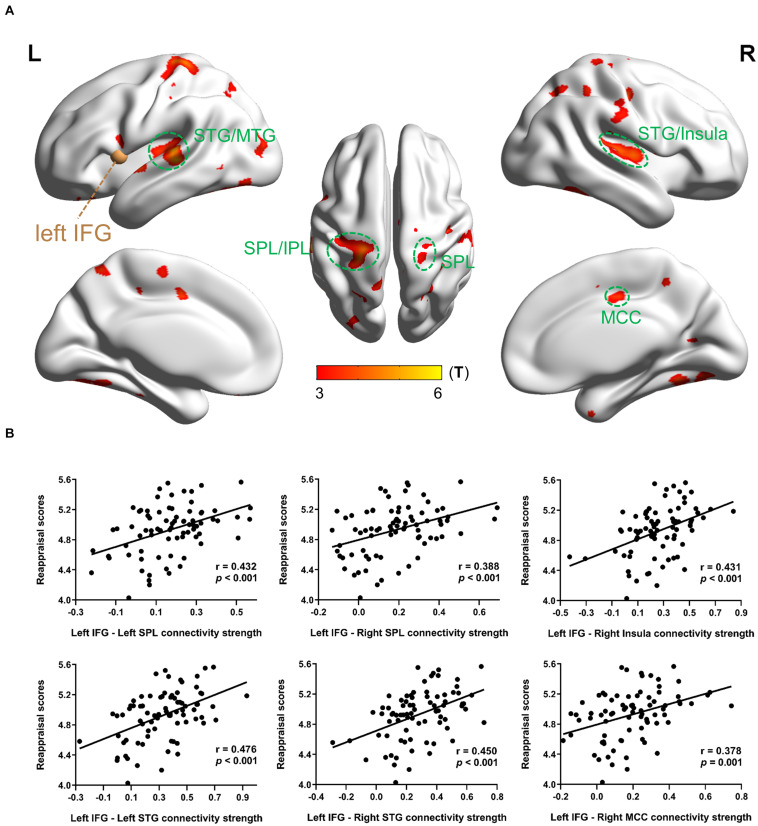
Results of the left IFG-based rsFC related with dispositional use of reappraisal in MRG. **(A)** Brain regions whose rsFC correlated with reappraisal scores. The brown sphere represented the seed region, and the green circles were drawn to display the ROIs. **(B)** Partial regression scatter plots depicted the correlation between functional connectivity strength and reappraisal scores. Statistical threshold of false discovery rate *P*_*FDR*_ < 0.05 was used for cluster correcting.

In MRG, with the left IFG as a seed region, reappraisal scores were positively correlated with rsFC between left IFG and most bilateral regions consisting of STG/MTG/ITG, superior parietal lobe (SPL), middle cingulate cortex (MCC), postcentral/precentral gyrus, rolandic operculum, cerebellum, and fusiform gyrus; and between left IFG and some left regions including inferior parietal lobe (IPL), supplementary motor area (SMA), precuneus, and occipital gyrus, as well as between left IFG and right insula (Table 4 and Figure 2A). However, there was no significant activation at the same threshold level in HRG.

**TABLE 4:** Functional connectivity results.

Region	H	K	T	MNI coordinates		
				*x*	*y*	*Z*
**Left IFG as a seed**						
Superior temporal gyrus/middle temporal gyrus/rolandic operculum	L	320	5.50	−57	−30	3
			5.26	−57	−27	12
Postcentral gyrus/precentral gyrus/superior parietal lobe/inferior parietal	L	949	5.09	−21	−36	66
lobe/middle cingulate cortex/supplementary motor area/precuneus			4.64	−21	−51	51
Cerebellum/fusiform/inferior temporal gyrus	R	314	5.01	24	−54	−18
			4.59	39	−54	−15
			4.46	48	−48	−21
Superior temporal gyrus/middle temporal gyrus/rolandic operculum/insula	R	562	4.81	72	−30	0
			4.57	39	−12	6
			4.49	63	3	6
Postcentral gyrus/precentral gyrus/superior parietal lobe	R	320	4.61	21	−36	66
Cerebellum/fusiform/inferior temporal gyrus	L	385	4.68	−42	−57	−24
			4.42	−39	−66	−18
Middle occipital gyrus	L	128	4.65	−30	−78	12
**Right IFG as a seed**						
Superior medial frontal gyrus/anterior cingulate cortex	L	294	5.45	−6	51	18
Cerebellum/fusiform/superior occipital gyrus/hippocampus/middle temporal gyrus/inferior temporal gyrus	L	1560	5.00	−36	−33	−27
			4.71	−30	−9	−21
Superior temporal gyrus/middle temporal gyrus	R	136	4.19	57	−42	21
Superior temporal gyrus/middle temporal gyrus	L	299	4.43	−45	−45	12
			3.88	−48	−57	6
Precuneus	L	111	4.40	−9	−54	45
Superior medial frontal gyrus/superior frontal gyrus	L	158	4.35	−6	36	51
	R		3.87	3	39	48
Superior occipital gyrus	R	152	4.09	15	−90	21
Middle temporal gyrus/insula	L	114	4.11	−60	−15	−9
			4.07	−45	−9	3
Postcentral gyrus/Precuneus	R	101	3.92	27	−39	51
			3.47	12	−54	45
Supramarginal gyrus/postcentral gyrus	L	144	3.66	−54	−27	30
			3.61	−54	−12	24

*H, hemisphere; L, left; R, right; K, cluster size in number of activated voxels; T, T value; MNI, the Montreal Neurological Institute coordinates. Statistical threshold of false discovery rate *P*_*F*__*DR*_ < 0.05 was used for cluster correcting.*

**FIGURE 3:**
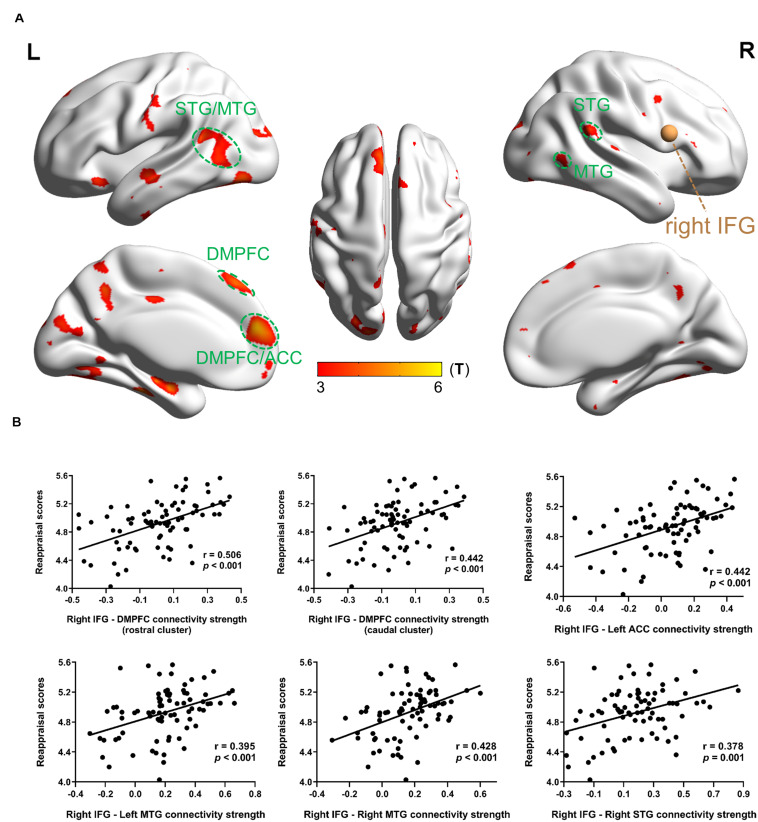
Results of the right IFG-based rsFC related with dispositional use of reappraisal in MRG. **(A)** Brain regions whose rsFC correlated with reappraisal scores. The brown sphere represented the seed region, and the green circles were drawn to display the ROIs. **(B)** Partial regression scatter plots depicted the correlation between functional connectivity strength and reappraisal scores. Statistical threshold of false discovery rate *P*_*FDR*_ < 0.05 was used for cluster correcting.

Functional connectivity analysis with right IFG as a seed region in MRG showed that reappraisal scores were positively correlated with rsFC between right IFG and brain areas such as the bilateral medial SFG, bilateral STG/MTG/ITG, bilateral precuneus, bilateral postcentral, bilateral occipital gyrus, bilateral fusiform, left ACC, left SFG, left insula, and right supramarginal gyrus (Table 4 and Figure 3A). Although in HRG, there were still no significant activation yielded at the same threshold level.

### Association of rsFC Strength With Reappraisal

When considering the left IFG as a seed region in MRG, the following ROIs showed significant correlation between strength values and reappraisal scores: left SPL (−21 −57 48), *r* = 0.432, *p* < 0.001; right SPL (24 −60 51), *r* = 0.388, *p* < 0.001; right insula (42 −12 6), *r* = 0.431, *p* < 0.001; left STG (−57 −27 12), *r* = 0.476, *p* < 0.001; right STG (54 −21 6), *r* = 0.450, *p* < 0.001; right MCC (9 −15 42), *r* = 0.378, *p* = 0.001 (Figure 2B).

On the other hand, when the right IFG is considered as a seed region in MRG, the following ROIs showed significant correlation between strength and reappraisal scores: DMPFC (rostral cluster) (−6 51 18), *r* = 0.506, *p* < 0.001; DMPFC (caudal cluster) (−6 36 51), *r* = 0.442, *p* < 0.001; left ACC (−6 48 12), *r* = 0.442, *p* < 0.001; left MTG (−48 −57 6), *r* = 0.395, *p* < 0.001 right MTG (42 −60 3), *r* = 0.428, *p* < 0.001; right STG (57 −42 21), *r* = 0.378, *p* = 0.001 (Figure 3B).

### Prediction Analysis

**FIGURE 4:**
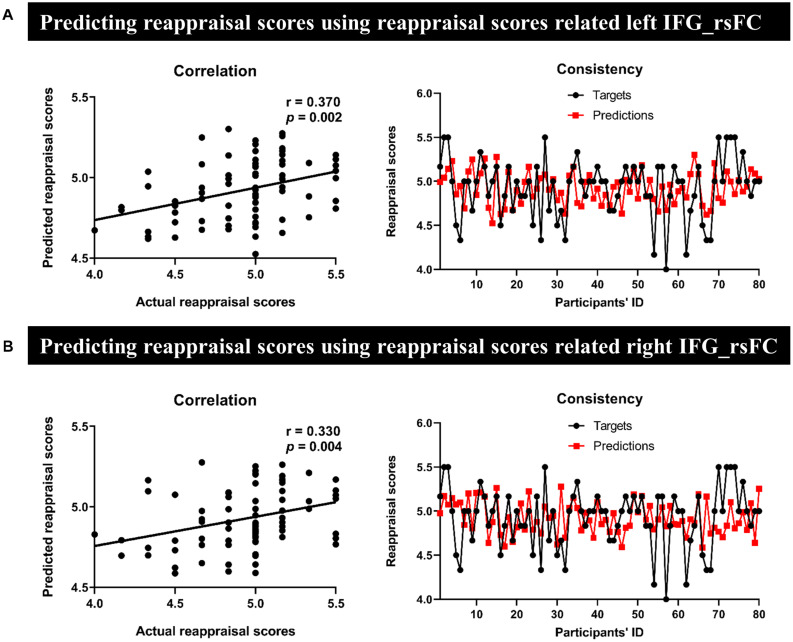
Results of prediction in MRG. Severally using **(A)** the left and **(B)** the right IFG-based rsFC to predict reappraisal scores. The scatter plots and line charts [in both panels **(A,B)**] described a significant correlation and consistency between actual and predicted reappraisal scores, respectively.

**FIGURE 5:**
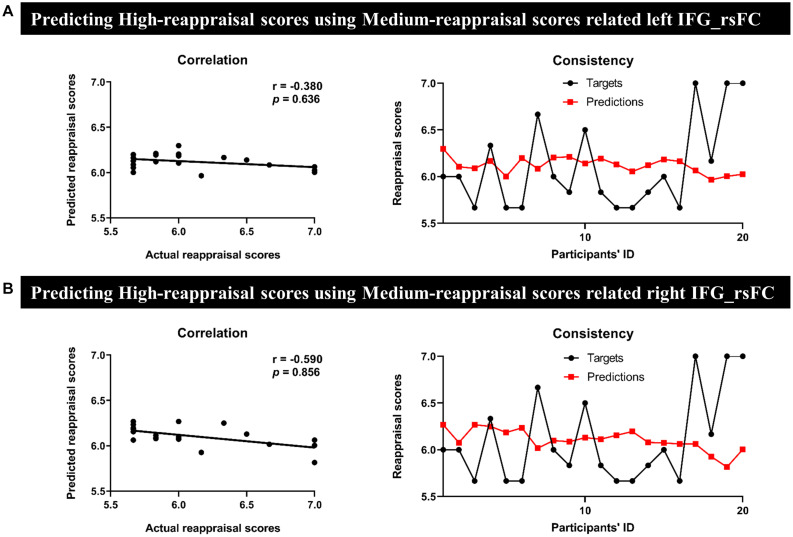
Results of prediction between HRG and sMRG. We separately used **(A)** the left and **(B)** the right IFG-based rsFC of sMRG to predict the reappraisal scores of HRG. Scatter plots and line charts indicated non-significant correlation and consistency between actual and predicted reappraisal scores, respectively.

In MRG, the left IFG seed-region-related rsFC could effectively predict for individual reappraisal scores (*r* = 0.370, *p* = 0.002) (Figure 4A), and this was also true for the right IFG seed region (*r* = 0.330, *p* = 0.004) (Figure 4B). However, there was no significant predictive power for individual reappraisal scores of HRG with left IFG or right IFG seed-region-related rsFC in sMRG (left IFG: *r* = −0.380, *p* = 0.636, Figure 5A; right IFG: *r* = −0.590, *p* = 0.856, Figure 5B).

## Discussion

Previous studies have emphasized the key functional role of IFG in collaboration with other regions in emotional regulation task-based functional connectivity, while the present study constitutes the first investigation into the associations between rsFC patterns of bilateral IFG and the dispositional use of reappraisal. Interestingly, we observed that medium dispositional use of reappraisal was positively related to IFG-based rsFC. Specifically, the medium habitual use of reappraisal was associated with a significant positive coupling between: (1) bilateral IFG and temporal gyrus; (2) left IFG and bilateral SPL, MCC, left IPL, and right insula; and (3) right IFG and DMPFC/ACC. However, no significant correlation emerged between left or right IFG-related rsFC and the high dispositional reappraisal use. The predictive analyses also showed that both left and right IFG-related rsFC could effectively and separately predict for individual reappraisal scores in MRG. However, IFG-related rsFC of sMRG had no significant predictive power for reappraisal scores of HRG.

In line with our hypotheses, the associations of both left and right IFG with temporal gyrus were linked to the habitual use of reappraisal. Particularly, using the reappraisal strategy altered the emotional value of stimuli by manipulating the conceptual knowledge and creating opposite interpretations. This suggests that the involvement of the semantic process is a core part of emotion regulation. Evidently, the semantic system plays a critical role in the storage and controlled retrieval of conceptual knowledge (Binder et al., 2009), contributing to representations of relevant emotional information from emotional experiences (Neumann and Lozo, 2012). Specifically, the temporal gyrus, which has often been observed with enhanced activation in reappraisal task (Goldin et al., 2008; Kanske et al., 2011; Dörfel et al., 2014), is usually considered as part of semantic system (Binder et al., 2009) and plays a role in both the storage and the strategic retrieval of semantic knowledge (Davey et al., 2016). Recent evidence on functional connectivity between IFG and MTG at both task (Zhang et al., 2019) and resting-state contexts (Kohn et al., 2014; Davey et al., 2016) indicates that the cooperation between IFG and MTG makes strategic access of semantic information possible. Similarly, the present study also revealed strong functional connectivity between IFG and temporal gyrus, alongside a positive correlation with medium habitual use of reappraisal, perhaps supporting the capacity to potentially engage and sustain semantic retrieval, in line with goal-driven control of subjective emotional feelings.

Inconsistent with our preliminary expectation, only the right IFG displayed an association with DMPFC. The DMPFC has generally been proved to be involved in semantic and self-reflective processes (Olsson and Ochsner, 2008; Binder et al., 2009), and has repeatedly been observed to be significantly activated in the reappraisal tasks (Kanske et al., 2011; Buhle et al., 2014; Morawetz et al., 2017a, 2016). In particular, DMPFC is implicated with the elaboration of the affective meaning of stimuli and representation of value information concerning mental states (Ochsner et al., 2012; Dixon et al., 2017). Thus, the right IFG, with a close connection with DMPFC, may facilitate the evaluation of the changing mental states, in relation to outcomes of appropriate or inappropriate interpretations of emotional stimuli. Besides, with a correlation with medium habitual reappraisal use, right IFG-based rsFC also showed a strong link with ACC. Recent evidence suggests that ACC constitutes a core part of the neural circuitry of valuation (Amemori and Graybiel, 2012; Bartra et al., 2013; Clithero and Rangel, 2013), playing an important role in evaluating interoceptive signals based on self-referential and conceptual emotion knowledge (Dixon et al., 2017). The evaluation role of ACC may thus facilitate a better understanding of subjective emotional feelings, by assigning conceptual meaning to these bodily sensations. Notably, through interaction with ACC, DMPFC contributes to the maintenance of mental representations of an individual’s feelings active in affective working memory (Lane et al., 2015) and may subsequently transfer these internal state information to IFG *via* a feed-forward mechanism (Phillips et al., 2008). Therefore, it is highly plausible that the right IFG (roles in selecting appropriate or inhibiting inappropriate interpretations from semantic memory) strongly connects with DMPFC and ACC (roles in perceiving and evaluating subjective emotional feelings), exhibiting correspondingly more frequent reappraisal skill, to achieve goal-directed emotional states.

Furthermore, as indicated above, we also observed a positive correlation between medium dispositional use of reappraisal and connectivity of left IFG with bilateral SPL, MCC, left IPL, and right insula during resting state. Consistent with the previous findings on the activation of parietal lobes in reappraisal task (Buhle et al., 2014; Morawetz et al., 2017b), our study also found an association between habitual use of reappraisal and co-activation of left IFG and parietal lobes (including SPL and IPL), which usually engage in the attention control process (Luks et al., 2007; Hutchinson et al., 2014; Salo et al., 2017; Sang et al., 2017; Wang et al., 2020). More importantly, it is documented that the parietal lobes and prefrontal gyrus engage in cognitive control by exerting influence on the temporal regions to change the semantic and perceptual representations, so as to facilitate the selection of appropriate behaviors, and the inhibition of maladaptive habitual actions (Buhle et al., 2014; Dixon, 2015). Similarly, MCC has also been found to be strongly involved in the allocation of attention to emotional information and action monitoring (McRae et al., 2008; Kragel et al., 2018). Indeed, the cognitive function of MCC in performance monitoring may help guide the changing emotional responses through reappraisal strategy in an intended way (Ochsner et al., 2012). Moreover, MCC also combines with the insula, which contains bodily information and sensations (including interoceptive representation of emotions) (Craig, 2009; Lane et al., 2015), to project the affective information to the neighboring IFG/VLPFC (Craig, 2009; Kohn et al., 2014), indicating a motivation to IFG to select an appropriate response in the final stage. During this process, IFG may work in concert with the parietal lobes and temporal gyrus to focus attention on the subjective feelings and select appropriate interpretation to obtain a desirable emotional state.

As initially anticipated, we did not observe a significant correlation between left or right IFG-related rsFC and the high dispositional reappraisal use. More so, the IFG-related rsFC pattern of MRG could not effectively predict for reappraisal scores of HRG. This supports the neural efficiency view, that the more adept the skill, the lesser the neural connectivity, but the more enhanced the performance becomes (Neubauer and Fink, 2009; Di Domenico et al., 2015; Curtin et al., 2019). However, there is a conspicuous lack of evidence on trait emotion regulation with reappraisal disposition to support our findings. Nevertheless, some promising evidence from cognitive training provides a potential explanation. For example, Vartanian et al. (2013) proved that working memory training can augment performance on divergent thinking task and lead to lower activation in the prefrontal gyrus (VLPFC and DLPFC) in adult participants, while Motes et al. (2018) found that cognitive training results in a faster processing speed along with reduced activation in the prefrontal gyrus in elderly participants. Therefore, it is possible that individuals with high-frequency daily use of reappraisal strategy, possess a more adept skill of emotion regulation, and recruit less functional connectivity between IFG and other regions in the resting state. On the other hand, the limited sample size in HRG may also be a potential reason to explain the non-significant results of both the association between rsFC and reappraisal scores, and the correlation in the internal cross-validation analysis. If so, future study should bring into consideration the sample size factor to ascertain whether the IFG-based rsFC pattern is linked to the variation of frequency level of dispositional use of reappraisal.

Overall, previous studies have emphasized the important role of the IFG in cooperation with other brain regions in task-based functional connectivity, in the selection and inhibition processes of emotional regulation. The present study expands on these findings by explicitly investigating how patterns of functional connectivity between IFG and other brain regions change during resting state, and how these changes may be linked to individuals’ habitual reappraisal use. Specifically, besides coupling with temporal gyrus in the function of general semantic control, we found that the left IFG, along with SPL, MCC, left IPL and right insula, predominantly engages in monitoring the emotional performance (cognitive control of emotion), while the right IFG, coupling with DMPFC and ACC, predominantly engages in representation of mental states (evaluation of emotion).

Despite the important contributions of our study, there are several limitations needed to be noted. Firstly, as mentioned above, the sample size of HRG is relatively smaller than that of the MRG. Possibly, there may be some other potential intrinsic functional connectivity patterns recruited by individuals with higher emotion regulation capacity. To fully understand the neural substrates of emotion regulation, more participants with a higher ability of emotion regulation need to be included in future studies. Secondly, the present study only recruited healthy participants. Although the neural association observed in these participants may provide potential neural evidence for clinical practice, the dispositional reappraisal use may show a different association with the resting-state networks in the context of emotional dysregulation. Therefore, future studies should consider a comparative analysis of the relevance of habitual strategy use and regulation networks, between patients with emotional disorders and healthy populations, to improve the clinical understanding and intervention. Finally, although we have not observed a significant association between IFG-based rsFC and suppression scores, which is also a personality trait of emotion regulation, it may exist in other potential functional hubs and neural substrates relevant to the dispositional use of the suppression strategy. Thus, future studies should explore the potential neural mechanism underlying trait suppression in both healthy and clinical populations.

In conclusion, the present study investigated the intrinsic neural underpinnings of dispositional reappraisal employing the IFG-based function connectivity approach during resting state. Our findings demonstrate that the medium dispositional reappraisal use relies on the cooperation of the functional hubs of the bilateral IFG and other regions within the emotion regulation cortex. These findings may explain how individuals cope with emotional events in daily life, as well as applied in clinical intervention for emotion-regulation-related disorders.

## Data Availability Statement

The original contributions presented in the study are included in the article, further inquiries can be directed to the corresponding authors.

## Ethics Statement

The studies involving human participants were reviewed and approved by the Local Committee for the Protection of Human Subjects of the University of Electronic Science and Technology of China. Written informed consent to participate in this study was provided by the participants.

## Author Contributions

WL, JZ, ZJ, and LL conceived and designed the experiments. WL performed the experiments. WL and KX analyzed the data. WL wrote the main manuscript text. RN revised the manuscript. All authors reviewed the manuscript.

## Conflict of Interest

The authors declare that the research was conducted in the absence of any commercial or financial relationships that could be construed as a potential conflict of interest.

## Footnotes

1 http://rfmri.org/DPARSF

2 http://www.fil.ion.ucl.ac.uk/spm

3 http://marsbar.sourceforge.net

4 www.mlnl.cs.ucl.ac.uk/pronto/
